# Dataset of Swimming behavioral alterations in *Danio rerio* by *Nemopilema nomurai* jellyfish venom

**DOI:** 10.1016/j.dib.2021.106721

**Published:** 2021-01-08

**Authors:** Ramachandran Loganathan Mohan Prakash, Du Hyeon Hwang, Il-Hwa Hong, Jinho Chae, Changkeun Kang, Euikyung Kim

**Affiliations:** a College of Veterinary Medicine, Gyeongsang National University, Jinju 52828, Korea; b Institute of Animal Medicine, Gyeongsang National University, Jinju 52828, Korea; c Marine Environmental Research and Information Laboratory, B1101, 17 Gosan-ro 148beon-gil, Gunpo-si, Gyeonggi-do 15850, Korea

**Keywords:** *Nemopilema nomurai* venom, *Danio rerio*, Intraperitoneal injection, Swimming behavior, Mortality rate

## Abstract

This article reports data associated with Prakash et al. [1]. Nemopilema nomurai jellyfish venom (NnV) can lead to neurotoxicity in zebrafish (Danio rerio) model*.* In the present study, zebrafish were treated with NnV by intraperitoneal injection and the swimming behavior of each fish was evaluated using a score scale. The dose of NnV in each treatment group was based on the protein concentration of NnV. Swimming is the main locomotory movements in the fishes. NnV modulated the swimming behavior of *Danio rerio* in a dose-dependent manner. In this article provided data are directly related to the previously published research article – “*Danio rerio* as an alternative vertebrate model for jellyfish venom study: the toxinological aspects of *Nemopilema nomurai* venom” [1] where the downregulation of acetylcholinesterase activity as well as histopathological alterations were observed from the brain of *Danio rerio* treated with NnV. Here we provide datasets, including mortality rate table, swimming behavior graph, and videos of zebrafish after NnV envenomation.

**Specifications Table**

**Table:** 

Subject	Biology
Specific subject area	Toxicology
Type of data	Tables, figure and videos
How data were acquired	*Danio rerio* as experimental model: dosage fixation based on mortality rate, swimming behavior of zebrafish after NnV treatment with different doses which includes 0, 10, 30, 100, 300, 500, 700, 900, 1100 and 1300 µg/g intraperitoneal injection.
Data format	Analyzed (Graph) Raw (Video)
Parameters for data collection	Dosage fixation data: quantity of live fishes (Mortality rate). Swimming behavior data: based on the locomotor movement of zebrafish.
Description of data collection	Mortality table data: zebrafish treated with NnV by intraperitoneal injection and monitored for 24 hours and accordingly mortality rate was calculated and represented in table. Swimming behavior data: The locomotor activity has evaluated through swimming behavior of NnV treated zebrafish and score scale is given.
Data source location	Gyeongsang National University, Jinju, South Korea. *Nemopilema nomurai* jellyfish specimens were collected from the Yellow Sea near the coast of Gunsan, South Korea (35.9677 ° N, 126.7366 ° E).
Data accessibility	With the article
Related research article	R.L.M. Prakash, D.H. Hwang, I.H. Hong, J. Chae, C. Kang, E. Kim, *Danio rerio* as an alternative vertebrate model for jellyfish venom study: the toxinological aspects of *Nemopilema nomurai* venom, Toxicology Letters (2020) (https://doi.org/10.1016/j.toxlet.2020.10.012).

## Value of the Data

• The data contribute to a better understanding the wide range of toxinological effects of NnV using Danio rerio; thereby may help the development of an effective first-aid treatment against poisonous jellyfish envenomation.
• The biochemical and histopathological approach in the present study will be useful for the researchers to develop a methodology and investigate neurotoxins using zebrafish animal model.
• Especially, when the amount of a natural toxin is so small, our zebrafish model suggests an alternative method for in vivo animal study, which would otherwise not be possible with conventional rodent model.
• Additionally, zebrafish model can be useful for the investigation of marine animal venoms, including jellyfish venom, in terms of illucidating its toxicological effects on aquatic animal species.

## 1 Data Description

**Fig. 1:**
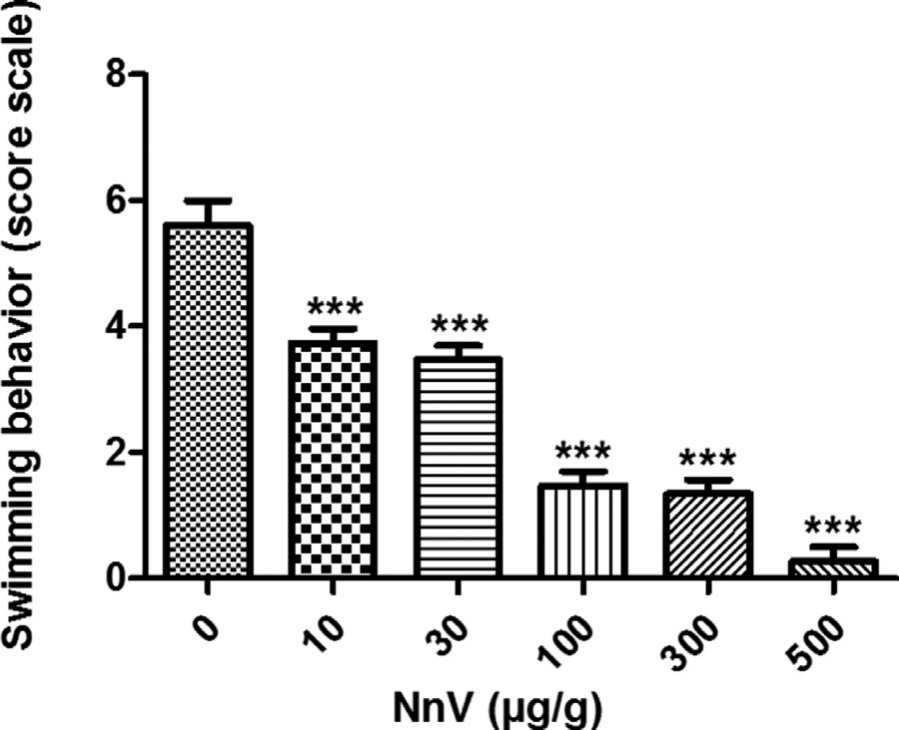
Effects of NnV on the swimming behavior of *Danio rerio*. The results are expressed as mean ± standard deviation (S.D.). The data were analyzed by one-way analysis of variance (ANOVA) followed by Dunnett's test to assess the significance of differences between control and treated group. The asterisks indicate significant differences (***: P<0.0001).

Table 1 describes the mortality rates of zebrafish intraperitoneally administered with various doses of NnV. All the zebrafish treated with 10 µg/g survived, whereas doses from 20 µg/g and above resulted in mortality, which increased in a dose-dependent manner. In addition, 900 µg/g of NnV and above resulted in 100% lethality. Additionally, Table 2 describes the score scale of swimming behavior of zebrafish. The swimming behavior (video 1) show the *Danio rerio* exposed to NnV displayed poor locomotor behavior, which was particularly notable at 100 and 300 µg/g doses. The “no movement” effect was observed at 500 µg/g dose. Fig. 1 depicts the swimming behavioral changes of zebrafish. The raw data are supplemented for the mortality rate and swimming behavior score scale of NnV treated *Danio rerio.*

**Table 1:** Mortality rate of NnV-treated *Danio rerio* through intraperitoneal injection.

Dose (µg/g)	Dead / Total no. of fishes	Mortality rate (%)
0	0/15	0
10	0/15	0
30	3/15	20
100	6/15	40
300	6/15	40
500	7/15	46.6
700	9/15	60
900	15/15	100
1100	15/15	100
1300	15/15	100

**Table 2:** Swimming behavior score scale of *Danio rerio.*

Score scale	Swimming behavior
0	Horizontal motionless position on bottom tank maintained for 2–3 min at a time at the peak of the narcosis and broken by a very brief change of position employing a slight stimulus
2	slowed swimming, normal body position
4	normality state
6	accelerated swimming, normal body position
8	frenetic swimming, with the body suspended in the vertical or some angled from the vertical
10	frenetic circling behavior

## 2 Experimental Design, Materials and Methods

### 2.1 Jellyfish nematocyst collection and preparation

*Nemopilema nomurai* jellyfish samples were collected from Yellow Sea near Gunsan coast, South Korea. The tentacles dissected from jellyfish were transferred instantaneously in ice to the laboratory for further processing steps. Briefly, dissected tentacles were rinsed with ice-cold seawater (4 °C) for removal of debris, and then added fresh seawater to the tentacle samples. They were swirled in shakers for 24 hours at 4 °C (autolysis). The supernatant (tentacle-free) was collected and centrifuged at 1000 × g for 5mins for harvesting nematocysts. The remaining sediments were further autolyzed and the harvesting procedure was repeated for additional 3-4 days as mentioned above. Finally, the nematocyst samples were spun at 500 × g for 5mins and the pellet (nematocyst-rich) was lyophilized. It is then stored at −20 °C for further use*.*

### 2.2 Venom extraction and preparation

Jellyfish nematocyst venom extraction is based on Carrette and Seymour [4]. with a slight modification. In brief, 50 mg of nematocyst is added with glass beads (approximately 8000 beads; 0.5 mm in diameter), and 1 ml of ice-cold phosphate-buffered saline (PBS, pH 7.4) as an extraction buffer. It is then, vigorously shaken on mini bead mill at 3000 rpm for 30 s. This extraction step was repeated 10 times with intermittent cooling on ice. The extracts were transferred to a new tube and spun at 22,000 × g at 4 °C for 30 min. The supernatant was used as NnV for further experiments. The NnV dose was determined based on protein concentration obtained by Bradford assay (Bio-Rad, Hercules, CA, USA).

### 2.3 Experimental design

All the fishes were acclimatized for one week before the experiment. Each experiment consists of 5 fishes / group. NnV was administered to zebrafish through intraperitoneal injection from 0 to 1300 µg/g of body weight. The mortality rates were calculated and exhibited in Table 1. Based on the mortality rate, the treatment dosages were determined, such as 0, 10, 30, 100, 300 and 500 µg/g. The swimming behavioral alterations were evaluated based on the score scale (Table 2) and presented in Fig. 1 and Video 1. All the experiments were repeated three separate times.

### 2.4 Dosage fixation upon mortality rate

According to Novak et al. [6], zebrafishes were weighed and anaesthetized in ice-cold water for approximately 2 min. Then, they were intraperitoneally (IP) injected with various dosages of NnV (10, 30, 100, 300, 500, 700, 900, 1100 and 1300 µg/g of body weight, respectively), according to Kinkel et al. [5]. The vehicle control group was injected with extraction buffer alone without NnV. Based upon the observation up to 24 hours of NnV treatment, mortality rate was determined (Table 1). The doses of 0 - 500 µg/g of NnV were chosen for the present toxinological study of the jellyfish venom.

### 2.5 Swimming behavior

Immediately after the NnV treatment, each fish was placed in a rectangular observation chamber (26 × 12 cm) containing home tank water filled at a level of 10 cm. There are totally five fishes (n=5) used per group in the experiment, which was separately repeated 3 times. Each fish was observed for 30s with 5 min of interval after NnV injection. The swimming behavior of each score scale (Table 2) was based on the method applied to Siamese fighting fish by Abramson and Evans [2] which is modified to *Danio rerio* according to Braida et al. [3]. According to the score scale, the swimming behavior of NnV-treated fishes were evaluated (Fig. 1).

### 2.6 Statistical analysis

The results are expressed as mean ± standard deviation (S.D.). The data were analyzed by one-way analysis of variance (ANOVA) followed by Dunnett's test to assess the significance of differences between control and treated group. The asterisks indicate significant differences (***: P<0.0001).

## Ethical Statement

All experiments comply with the ARRIVE guidelines and were be carried out in accordance with the U.K. Animals (Scientific Procedures) Act, 1986 and associated guidelines, EU Directive 2010/63/EU for animal experiments, or the National Institutes of Health guide for the care and use of Laboratory animals (NIH Publications No. 8023, revised 1978). All the experiments performed based on the Regulations for Animal Care was approved by the Ethical Committee of Gyeongsang National University according to the protocol number GNU-200519-E0028.

## Declaration of Competing Interest

The authors declare that they have no known competing financial interests or personal relationships, which have, or could be perceived to have, influenced the work reported in this article.

## Appendix Supplementary materials

**Video 1.** Swimming behavioral alterations of *Danio rerio* upon the treatment of NnV in the range of 0, 10, 30, 100, 300 and 500 µg/g, respectively. The score scale of each fish was determined based on Abramson and Evans (1954). Image, application 1

Image, application 2
